# Investigation of Adiposity Measures and Operational Taxonomic unit (OTU) Data Transformation Procedures in Stool Samples from a German Cohort Study Using Machine Learning Algorithms

**DOI:** 10.3390/microorganisms8040547

**Published:** 2020-04-10

**Authors:** Martina Troll, Stefan Brandmaier, Sandra Reitmeier, Jonathan Adam, Sapna Sharma, Alice Sommer, Marie-Abèle Bind, Klaus Neuhaus, Thomas Clavel, Jerzy Adamski, Dirk Haller, Annette Peters, Harald Grallert

**Affiliations:** 1 Research Unit of Molecular Epidemiology, Helmholtz Zentrum München, 85764 Neuherberg, Germany; stefan.brandmaier@helmholtz-muenchen.de (S.B.); jonathan.adam@helmholtz-muenchen.de (J.A.); sapna.sharma@helmholtz-muenchen.de (S.S.); 2 Institute of Epidemiology, Helmholtz Zentrum München, 85764 Neuherberg, Germany; ajsommer@fas.harvard.edu (A.S.); peters@helmholtz-muenchen.de (A.P.); 3 ZIEL Institute for Food & Health, Technical University of Munich, 85354 Freising-Weihenstephan, Germany; sandra.reitmeier@tum.de (S.R.); neuhaus@tum.de (K.N.); tclavel@ukaachen.de (T.C.); dirk.haller@tum.de (D.H.); 4 Chair of Nutrition and Immunology, Technical University of Munich, 85354 Freising-Weihenstephan, Germany; 5 Department of Statistics, Faculty of Arts and Sciences, Harvard University, Cambridge, MA 02138-2901, USA; ma.bind@mail.harvard.edu; 6 Functional Microbiome Research Group, Institute of Medical Microbiology, RWTH University Hospital, 52074 Aachen, Germany; 7 Research Unit Molecular Endocrinology and Metabolism, Helmholtz Zentrum München, 85764 Neuherberg, Germany; adamski@helmholtz-muenchen.de; 8 Chair of Experimental Genetics, Technical University of Munich, 85350 Freising-Weihenstephan, Germany; 9 Department of Biochemistry, Yong Loo Lin School of Medicine, National University of Singapore, Singapore 117597, Singapore; 10 Chair of Epidemiology, Faculty of Medicine, Ludwig-Maximilians-University München, 81377 Munich, Germany; 11 German Center for Diabetes Research (DZD), 85764 Neuherberg, Germany

**Keywords:** gut microbiota, obesity, machine learning, waist–height ratio, 16S rRNA

## Abstract

The analysis of the gut microbiome with respect to health care prevention and diagnostic purposes is increasingly the focus of current research. We analyzed around 2000 stool samples from the KORA (Cooperative Health Research in the Region of Augsburg) cohort using high-throughput 16S rRNA gene amplicon sequencing representing a total microbial diversity of 2089 operational taxonomic units (OTUs). We evaluated the combination of three different components to assess the reflection of obesity related to microbiota profiles: (i) four prediction methods (i.e., partial least squares (PLS), support vector machine regression (SVMReg), random forest (RF), and M5Rules); (ii) five OTU data transformation approaches (i.e., no transformation, relative abundance without and with log-transformation, as well as centered and isometric log-ratio transformations); and (iii) predictions from nine measurements of obesity (i.e., body mass index, three measures of body shape, and five measures of body composition). Our results showed a substantial impact of all three components. The applications of SVMReg and PLS in combination with logarithmic data transformations resulted in considerably predictive models for waist circumference-related endpoints. These combinations were at best able to explain almost 40% of the variance in obesity measurements based on stool microbiota data (i.e., OTUs) only. A reduced loss in predictive performance was seen after sex-stratification in waist–height ratio compared to other waist-related measurements. Moreover, our analysis showed that the contribution of OTUs less prevalent and abundant is minor concerning the predictive power of our models.

## 1. Introduction

As a consequence of modern lifestyles, excessive body weight has become an important health burden, with a prevalence that has greatly surpassed underweight on a global scale [1]. Understanding biological mechanisms associated with obesity in combination with changes in the daily habits and living conditions of people will help address this challenge of current health systems and societies. One of the biological factors influencing obesity is the gut microbiome [2], which has also been linked to comorbidities, such as diabetes [3], and to even further pathophysiological processes, such as neurological and mental disorders [4]. These associations seem not surprising as extensive research has shown many axes between the gut microbiome and metabolically relevant organs such as the liver, adipose tissue, and even the brain [5]. Moreover, the gut microbiota has its role in the mediation and conversion of external input from the environment, including medication [6,7], food, and energy metabolism [5,8,9]. These findings introduced the gut microbiome as a key player for the comprehension of and possibly novel intervention strategies for the obese phenotype. However, there is no clear consensus on how the association between obesity and the gut microbiome is shaped [5,10,11]. For improved understanding of the interactions and derivation of correct conclusions, it is important to dissect data processing strategies and their impact on study outcomes [12,13].

Human gut microbiota are commonly analyzed using high-throughput 16S rRNA gene amplicon analysis of feces, an easily accessible and noninvasive sampling procedure for health assessments. Its ubiquity in bacteria and the combination of highly conserved and variable regions within the 16S rRNA gene makes it suitable for compositional analyses of the bacterial gut content [14]. During data processing, sequenced DNA fragments are usually clustered into operational taxonomic units (OTUs) based on sequence similarity above a determined threshold, though other analysis strategies are also emerging [15]. Despite the ability to rapidly obtain a comprehensive overview of microbiota profiles using amplicon sequencing, the resulting data is of inherent compositional nature due to technical instruments being limited in their maximum measurement capacities. The occurrence of OTUs is therefore expressed in relative abundances with inherent dependencies among them. In effect, an increase in counts measured of one OTU will cause a decrease in available measurement slots for other OTUs. However, it is assumed that the resulting counts reflect the underlying distribution in native samples to a high degree [12]. Further, many OTUs are found only in one or few samples, causing sparsity in the OTU table, i.e., great amounts of zeros, which poses an additional challenge in OTU data analysis [13].

Consequently, standard statistical procedures are likely not able to account for these constraints and lead to suboptimal data evaluation. Log-ratio transformations, in particular centered log-ratio (CLR) and isometric log-ratio (ILR), as preprocessing methods for OTU data have been discussed in coping with some of these issues, as they are invariable, whether they have been calculated from the measured count data or the unknown complete original sample. In the case of the CLR, the OTUs are displayed in relationship to the geometric mean of the sample, while ILR uses orthonormal bases [12,16]. 

In the present study, we evaluated the suitability of different methods to capture the complexity of a recently generated OTU data set in approx. 2000 individuals from the cross-sectional KORA (Cooperative Health Research in the Region of Augsburg) FF4 study (2013/14). Specifically, we investigated the practical consequences of data transformations in light of machine learning prediction. Moreover, we compared nine measures of obesity and how well they could be represented by the fecal microbiota profiles.

## 2. Materials and Methods 

### 2.1. Study Population

The analyses were based on participants’ data from the KORA (Cooperative Health Research in the Region of Augsburg) FF4 study. As the 2nd population-based follow-up examination of the KORA S4 study (1999/2001), it was conducted in 2013/2014 with 2279 individuals in southern Germany. The investigations were carried out in accordance with the Declaration of Helsinki, including written informed consent of all participants. All study methods were approved by the ethics committee of the Bavarian Chamber of Physicians, Munich (FF4: EC No. 06068, 25 October 2012) [17,18]. The informed consent given by KORA study participants does not cover data posting in public databases. However, data are available upon request from KORA-gen (http://epi.helmholtz-muenchen.de/kora-gen/) by means of a project agreement. Requests should be sent to kora.passt@helmholtz-muenchen.de and are subject to approval by the KORA Board.

### 2.2. Microbiota Profiling by 16S rRNA Amplicon Sequencing

The microbiome measurement procedures via high-throughput 16S rRNA gene sequencing have been described in detail elsewhere [19]. In brief, of all 2272 participants from the KORA FF4 study, individuals with missing consent were excluded (*n* = 197). The remaining participants were asked to collect a stool sample in a collection tube containing DNA stabilizer (Stratec DNA Stool Stabilizer, Cat. no. 1038111100), which were finally stored at −80 °C. After DNA extraction, the V3-V4 regions of the 16S rRNA gene were amplified via 2-step PCR (primers 341F-ovh and 785r-ovh) and sequenced on Illumina HiSeq. To guarantee high-quality output, samples with low read counts were resequenced on an Illumina MiSeq. Raw sequence reads were processed using the IMNGS platform [20] that integrates a UPARSE-based OTU clustering approach at 97% sequence similarity level [21]. Only OTUs occurring at a relative abundance ≥0.25% in at least one sample across the entire cohort study were kept to avoid the analysis of spurious taxa [22]. For technical quality control, samples with an insufficient amount of DNA content (<12 ng/μL) or reads (<7000) were excluded as well (*n* = 42). In total, 2089 OTUs were identified for the complete study with usable fecal samples.

### 2.3. Exclusion Criteria

Of the available 2033 individuals with fecal microbiome measurements, participants with antibiotic medication intake (*n* = 41), malignant neoplasms of digestive organs according to WHO ICD-10 codes C15-C26 (*n* = 27), and underweight with a BMI < 18.5 kg/m^2^ according to WHO criteria (*n* = 8) were excluded. All individuals with missing information in one of the adiposity measures were additionally excluded (*n* = 35), resulting in a study population of 1923 individuals.

### 2.4. Adiposity Measures

Nine adiposity measures were examined in this study: body mass index (BMI; kg/m^2^), waist circumference (WC; cm), waist–hip ratio (WHR), waist–height ratio (WHtR), body adiposity index (BAI; according to the formula by Reference [23]: (hip circumference (cm))/((height (cm) × 0.01)^1.5^) − 18), fat mass index (FMI; kg/m^2^), lean body mass index (LBMI; kg/m^2^), appendicular muscle mass index (AMMI; kg/m^2^), and body fat (BF; %). Body composition was measured via bioelectrical impedance analysis (BIA 2000-S device, Data Input, Pöcking, Germany) with Kyle’s equations [24,25,26,27]. To enable comparability of results, all adiposity measures were scaled to a mean of 0 and standard deviation of 1 for each (sub-)set for machine learning, separately.

### 2.5. Machine Learning Algorithms

In this study, four different machine learning algorithms, i.e., support vector machine regression (SVMReg) with normalized poly kernel (NPK), random forest (RF), and M5Rules as well as partial least squares (PLS) with different numbers of principal components, 20 (PLS20) and 4 (PLS4), were compared. All prediction models were calculated in Weka version 3.8.3, except for PLS in Weka version 3.6.15 [28,29], using default options except the following parametrizations: M5rules: minNumInstances 4.0; SVM: filter type no normalization/standardization; and NPK: E2.0. From the Weka summary statistics, the correlation coefficients (CC) were used for model evaluation. They represent the Pearson correlation between the actual and the predicted values of the outcome (i.e., obesity measurement) from 10-fold cross-validation.

### 2.6. Statistical Analysis

The remaining analyses were conducted in R version 3.6.0 [30]. For each (sub-)set of the data, data transformations were executed separately and each transformation was calculated sample-wise. Relative abundance (RA) was calculated with each sample sum to 100%. Logarithm (Log) refers to the natural logarithm. Centered log-ratio (CLR) and isometric log-ratio (ILR) were generated with the compositions package [31] in R. Raw count data was included for further analyses as the control value. Alpha diversity as Simpson effective counts according to Jost [32] was calculated via the vegan package in R [33]. For comparison of prediction rates with and without sparsely occurring OTUs, across-sample prevalence and abundance were calculated for the complete OTU data set and respective OTUs were excluded in a stepwise fashion. For prevalence, OTUs present in less than x% of the samples were excluded; for abundance, OTUs with a relative abundance equal to or less than x% were excluded. The term prevalence was used in this study to describe the number of times an OTU was nonzero in the samples.

## 3. Results and Discussion

### 3.1. Benchmark Data on Study Population and Adiposity Measures

**Figure 1:**
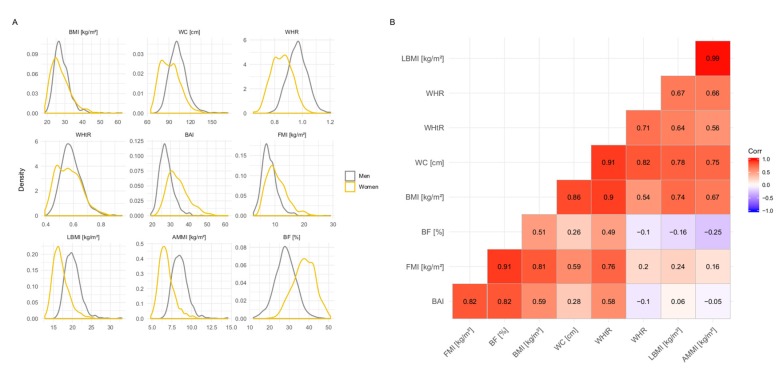
Comparison of adiposity measures: (**A**) Sex-stratified distribution of adiposity measures and (**B**) Spearman correlation in study population (*n* = 1923). Adiposity measurements were z-transformed (mean = 0, standard deviation = 1). AMMI: appendicular muscle mass index; BAI: body adiposity index; BF (%): body fat percentage; BMI: body mass index; FMI: fat mass index; LBMI: lean body mass index; WC: waist circumference; WHR: waist–hip ratio; WHtR: waist–height ratio.

First, we examined the features of nine adiposity measures within our study population. On average, study participants were older than the German median age [34] as well as overweight according to the World Health Organization (WHO) guidelines [35] (Table 1). Three different components of adiposity assessment were examined, including representation of overall obesity (i.e., BMI), body shape (i.e., waist circumference, waist–hip ratio, and waist–height ratio), as well as body composition measurements with its two subcomponents of muscle-related measures (i.e., lean body mass index and appendicular muscle mass index) and fat-related measures (i.e., fat mass index, body fat percentage, and body adiposity index). Each measure exhibited a large range, approximating a comprehensive coverage of possible forms for each phenotype, except underweight (Figure 1A and Table S1). For example, the lowest BMI measurement in our data was 18.6 kg/m^2^ (low normal weight) and the highest was at 62.2 kg/m^2^ (severely obese). 

On the topic of sex differences in adiposity measures, three aspects can be noted from the present data (Figure 1A): (i) The distribution of most measures related to body shape as well as body composition showed distinct sex-specific differences in their distribution. (ii) In certain measures, women showed dual peaks in their distribution of obesity, particularly in waist-related measurements. This was likely due to the simultaneous existence of android and gynoid fat distributions within adult female populations. The former might in part be due to menopause in a slightly older study population. This is in contrast to adult men with a predominant android phenotype [36,37]. (iii) On the other hand, accounting for height was able to reduce sex-related biases in some indices, such as in BMI and waist–height ratio.

Additionally, three aspects on the relation of adiposity measures among each other are set out from the comparison of Spearman correlations (Figure 1B): (i) The same two indices, BMI and waist–height ratio, both showed reasonable correlations to all other adiposity measurements, ranging from 0.51 to 0.90 for BMI and 0.49 to 0.91 for waist–height ratio. (ii) Closely related measurements, i.e., within one (sub-)component of adiposity assessment, demonstrated particularly high correlations: e.g., lean body mass index and appendicular muscle mass index (0.99), fat mass index and body fat percentage (0.91), or waist circumference and waist–height ratio (0.91). (iii) Furthermore, measurements from one closely related group sometimes exhibited negative correlations to measurements from another group, e.g., appendicular muscle mass index and body fat percentage (−0.25) or waist–hip ratio and body adiposity index (−0.10).

### 3.2. Adiposity Measurement Selection

We trained prediction models for obesity using adiposity measurements as outcome and OTU data as predictors. We calculated models for all possible combinations of body measurements, preprocessing procedures, and machine learning algorithms (Table 2). For evaluation, we used correlation coefficients (CCs) from cross-validation between actual and predicted values of the outcome (see Section 2, Materials and Methods), with a potential value of 1 at its best and of 0 at its worst (as a negative CC would indicate a model, which predicts the opposite of the measured values).

Our results revealed the lowest prediction rates for measurements related to body fat (i.e., body adiposity index, fat mass index, and body fat percentage). CCs for these measurements varied from 0.04 (body fat percentage in combination with relative abundance and M5Rules) to 0.28 (fat mass index and body fat percentage with support vector machine regression (SVMReg) and log-ratio transformations). By comparison, the highest prediction rates were achieved with measures related to central fat accumulation (i.e., waist circumference, waist–hip ratio, and waist–height ratio). These measurements were able to predict the variation in the phenotypes up to almost 40%. Additionally, our results showed that measurements related to muscle mass (i.e., appendicular muscle mass index and lean body mass index) were also well predicted by fecal OTU data. Specifically, the explained variation in muscle-related measurements of our study reached 36% and 35% for lean body mass index and appendicular muscle mass index, respectively.

These findings may lead to several indications. (i) Our data suggested a link between the gut microbiota profiles and central fat rather than body fat content in general. These findings are in line with previous studies that identified a stronger relationship of central obesity measurements than to other obesity measurements such as BMI. In particular, visceral fat has shown associations with the gut microbiome and this could be the reason behind the higher correlation towards waist measurements compared to other parameters. For example, Zierer et al. speculated that the association of the gut microbiome to fatty acid metabolism “may be better reflected by visceral fat measures” [38] as well as an interrelation with food to influence visceral fat [38,39,40,41]. (ii) The prediction capacity in muscle measurements supported recently published experiments in mice that demonstrated effects of the gut microbiota on the quantity, mass, and functional characteristics of skeletal muscles [42]. (iii) Taken together, these findings might suggest a link of gut microbiota composition and cardiometabolic health. Central obesity, for example, high waist–height ratio, has been identified as a risk factor for cardiovascular disease mortality [43], whereas muscle mass seems to protect from cardiovascular disease [44]. Additionally, previous studies already have shown associations of the gut microbiome with cardiometabolic risk factors [45] and are supported by mechanistic studies in mice, suggesting a role for gut bacteria in dietary energy harvest and fatty acid metabolism [41]. These findings should convey further investigations, examining the role of the gut microbiome in cardiometabolic health. Among other things, this observational study could be embedded in a hypothetical randomized experiment, as suggested by Bind and Rubin [46].

### 3.3. Comparison of OTU Data Transformation Approaches

Because of ongoing debates on optimal data normalization, transformation, and general preprocessing [13,47], we aimed to evaluate different methods within our data set and in combination with our target outcomes, i.e., obesity phenotypes (Table 2). Logarithmic transformations (i.e., sample-wise relative abundance with log-transformation, CLR, and ILR) generally improved the prediction performance independent of outcome or machine learning algorithm, except for random forest (RF). The best results were achieved with waist measurements, in particular in combination with SVMReg and CLR (optimal CCs for each waist measurement between 0.36 and 0.39) and PLS4 in all log-transformations (0.35–0.37). This was underlined when comparing RA values with and without a log-transformation. This ensured a distinction between different methods and the improvement resulting from employing a logarithmic transformation as such [48]. These results supported the assumption of logarithmic transformations as a useful tool in microbiota data analysis and from a machine learning perspective in particular.

Comparing the different transformation methods that include a logarithmic transformation, the results suggested only minor differences between the methods in terms of practical application to OTU data. Although CLR and ILR achieved the overall best results in combination with waist circumference, they did not lead to a big improvement in performance in comparison to log-transformed RAs. For our subsequent analyses with SVMReg, the easier interpretation of CLR values as a relation to the geometric mean made it preferable to ILR.

**Figure 2:**
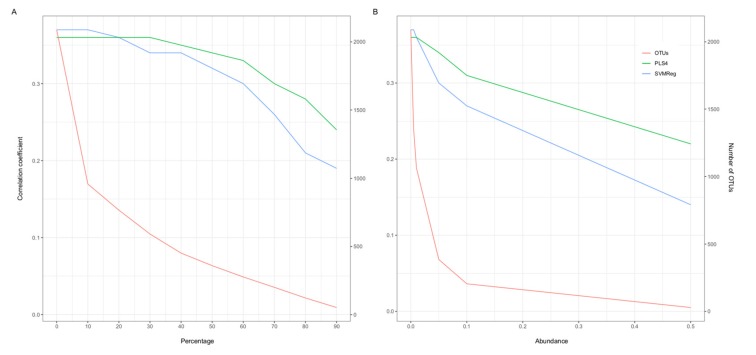
Dependency of prediction capacity on prevalent and abundant OTUs in waist–height ratio, implemented with the combinations SVMReg NPK with CLR, and PLS4 with RA+Log: In each step, (**A**) OTUs present in less than x% of the samples were excluded (prevalence); this means for example in the step 80%, that OTUs with a prevalence of less than 80% were excluded for the calculations. (**B**) OTUs with an across-sample relative abundance of x% or less were excluded (abundance).

Log-transformations have been critically discussed for their potential to increase the impact of less prevalent and abundant OTUs and vice versa for dominant OTUs in certain contexts [47]. Our analysis conversely suggests an improvement in performance with logarithmic transformations in most cases, with no emphasis on sparsely occurring OTUs in the prediction of waist–height ratio (Table 2, Table S2, and Figure 2). Moreover, log-transformations are able to manage skewedness and to limit the large variation in raw count data and already lead to major improvements in predictions in our data set. Therefore, we assumed that difficulties for many algorithms in coping with said variation, and in particular SVMReg and PLS4, benefited from log transformations. For example, for waist–height ratio predictions, the CC improved from 0.21 (SVMReg) and 0.23 (PLS4) in raw count data up to 0.37 (SVMReg) and 0.36 (PLS4) in logarithmically transformed data. In contrast, an algorithm based on randomness like random forest (RF) is able to work comparatively well with raw count data and RAs and even almost consistently better than with logarithmically transformed data. In the same example as before, RF achieved a correlation of 0.31 in raw count data but of 0.25 in CLR.

### 3.4. Implications from Machine Learning Algorithms

The juxtaposition of machine learning algorithms contained mixed messages. On the one hand, algorithms without assumptions on data structure, such as SVMReg and RF, showed good overall performance, which may underline the importance of accounting for the complex nature of OTU data. For instance, OTUs are clusters of 16S rRNA gene sequences using a similarity threshold without possible functional differentiation. Thus, an OTU may contain different strains of the same species or species which are taxonomically very close. Consequently, such groupings may contain strains even with opposite effects on a phenotype [49,50]. In addition, OTUs as representation of bacteria in the human gut likely do not exist as isolated entities but have many interrelations among each other [51,52]. In particular SVMReg, which transforms into higher dimensional feature spaces via the use of kernels, seemed well-suited to account for such interdependencies of OTUs. 

On the other hand, linear models, here exemplified by PLS, performed similarly well on the OTU data if used with a low number of principal components. This was in particular the case after logarithmic transformation, which lead to a normality-like distribution optimal for a linear approach. In this light, our data supports a linear dependency between logarithmically transformed OTU data and adiposity endpoints in our data set. 

The results concerning the number of principal components also raised the question of data overfitting. A high number of components (PLS20) consistently lead to worse performance, while a low number (PLS4) represented one of the best performing methods used. Therefore, the number of parameters contained in a linear model should be critically assessed and kept to a necessary minimum.

### 3.5. Effect of Sex-Stratification on Predictive Performance

In a next step, we examined prediction rates of abdominal measurements in a sex-stratified approach using two of the best performing combinations (i.e., SVMReg with CLR and PLS4 with log-transformed RAs). Of the three abdominal measurements compared in our study, waist–height ratio had the lowest decrease in predictive performance in the sex-stratified data sets in both methods. With PLS4, the CCs in waist–height ratio decreased from 0.36 in the total population to 0.28 and 0.34 in men and women, respectively. By comparison, with waist circumference the CCs dropped from 0.37 to 0.27 (men) and 0.31 (women). This indicates that the sex-specific variation in waist circumference-related measures could be reasonably addressed by accounting for height (Figure 1A and Table 3). Therefore, waist–height ratio combined the advantages of BMI, i.e., height adjustment and reasonable correlations to other adiposity measurements (Figure 1B), with the improved prediction capacity of waist measurements.

However, generally, it could be observed that the sex stratification reduced prediction capabilities. To attribute this effect to the smaller sample size seems implausible, as the sensitivity of machine learning, in contrast to *p*-values, is unlikely to be impaired in population sizes that vary in the magnitude of almost 1000 to 2000 (Table 1 and Table 3). Still, a data split could cause particularly sex-specific OTUs to be less prominent in either of the strata and, hence, considered to be less informative by the algorithm than in the complete data set. This should warrant specific attention to sex differences and appropriate analysis approaches for future studies.

However, the effects on the predictive performance were also distributed unevenly among the sexes, whereby the phenotypes are consistently better predicted in women. One reason for this might be the slightly narrowed distribution of waist–height ratio values in men compared to women (Figure 1A). Another factor might be the, on average, increased ecosystem diversity in women (Figure S1). In a previous publication on the dietary habits of the KORA FF4 population, Breuninger et al. showed that women consume a more fiber-dense diet than men [53] and, hence, a rather favorable nutrition for the gut microbiome linked to increased gut bacterial diversity.

### 3.6. Dependency of Prediction Capacity on Prevalent and Abundant OTUs

In order to analyze the contribution of low abundant/prevalent OTUs to the predictive performance of our models, we excluded OTUs in a stepwise fashion according to their across-sample prevalence and abundance in the data set. For this purpose, we used the same two combinations as methods (i.e., SVMReg with CLR and PLS4 with log-transformed RAs) to predict waist–height ratio. However, our results suggested that the prediction of waist–height ratio was mainly based on frequent and abundant OTUs (Figure 2 and Table S2). We observed the same prediction capacity after the exclusion of OTUs which occurred with a prevalence of less than 30% of the samples, which make up more than 72% of all OTUs in the data. Even after the exclusion of 98% of all OTUs, the top 52 entities were able to explain almost a quarter of the variation in waist–height ratio in the population as estimated by PLS4. Similar approaches have been implemented in previous studies, e.g., using a threshold of 25% [38,39]. Within our analyses of waist–height ratio, the same threshold would merely lead to a decrease in prediction capacity by approximately 0.01–0.03 with SVMReg and no decrease in PLS4. The same could be observed for across-sample relative abundance. An exclusion of OTUs with an abundance of 0.01% or less, which excluded close to 50% of OTUs, led to almost no (−0.01, SVMReg) to no decrease (PLS4) in correlation coefficients. In a scenario where the quality of prediction is mainly determined by prevalent and abundant OTUs, it seems plausible that the effects of pseudocounts, an added value to offset zeros before log-transformation, was only minimal as shown in our analyses (Table S3). This is relevant to the criticism of log-ratio transformations, in that they need coping mechanisms for the zero-inflation in OTU data sets and of which pseudocounts are one possible choice [13].

### 3.7. Limitations

This study has several limitations. First, the study population reflected a middle-aged to elderly part of the population (38–88 years, Table S1). Therefore, the findings cannot be extrapolated to all age groups. Moreover, causal relationships cannot be established in this kind of study, and further validation from experimental data is needed. In addition, the tests were restricted to a dataset from one cohort study, and therefore, these results on their own cannot be generalized. The cross-sectional nature of our study only permits the assessment of correlations of a specific point in time for the participants. Recent changes in weight cannot be excluded but should average out with sufficient sample size.

## 4. Conclusions

In summary, we applied a selection of preexisting methods to compare the relation between adiposity measures and OTU data for the KORA FF4 study specifically. We identified waist-related obesity measures to be reflected best by our OTU data. In particular waist–height ratio, which showed less bias in sex-stratification compared to the total population, seemed to be the most appropriate choice. Our analyses showed that almost 40% of the variance in abdominal obesity could be reflected by composition of fecal microbiota. The predictive ability is mostly guaranteed by prevalent and abundant OTUs. 

## Figures and Tables

**Table 1:** Benchmark data on the KORA (Cooperative Health Research in the Region of Augsburg) FF4 study population (mean (standard deviation)).

Characteristics	Overall (*n* = 1923)	Men (*n* = 936)	Women (*n* = 987)
Age (years)	60.0 (12.1)	60.3 (12.3)	59.7 (11.9)
Body mass index (kg/m^2^)	27.9 (5.0)	28.3 (4.5)	27.5 (5.4)
Waist circumference (cm)	97.0 (14.2)	102.8 (12.4)	91.5 (13.6)
Waist–hip ratio	0.91 (0.09)	0.96 (0.07)	0.85 (0.07)
Waist–height ratio	0.58 (0.08)	0.59 (0.07)	0.56 (0.09)
Body adiposity index	31.0 (6.0)	27.8 (4.0)	34.0 (5.9)
Fat mass index (kg/m^2^)	9.4 (3.4)	8.1 (2.8)	10.6 (3.5)
Lean body mass index (kg/m^2^)	18.5 (2.6)	20.1 (2.1)	16.9 (2.1)
Appendicular muscle mass index (kg/m^2^)	7.7 (1.3)	8.6 (1.0)	6.8 (1.0)
Body fat (%)	32.9 (7.1)	28.1 (5.3)	37.5 (5.5)

**Table 2:** Comparison of adiposity measures, transformation methods of operational taxonomic unit (OTU) count data, and machine learning algorithms in the study population (*n* = 1923): Each method includes 10-fold cross-validation, and for each logarithmic transformation, one pseudocount was added to the raw counts. CC: correlation coefficient; CLR: centered log-ratio transformation; ILR: isometric log-ratio transformation; NPK: normalized poly kernel; PLS: partial least squares regression; RF: random forest; RA: relative abundance; RMSE: root mean squared error; SVMReg: support vector machine regression.

Data Transformation	Machine Learning Algorithm									
	SVMReg NPK		RF		M5Rules		PLS20		PLS4	
	CC	RMSE	CC	RMSE	CC	RMSE	CC	RMSE	CC	RMSE
Body mass index										
Raw counts	0.18	1.01	0.26	0.97	0.19	1.06	0.17	1.24	0.21	0.99
RA (100%)	0.18	1.01	0.26	0.97	0.23	1.02	0.21	1.11	0.22	0.99
RA + Log	0.30	0.97	0.25	0.97	0.26	1.01	0.22	1.22	0.32	0.96
Raw counts + CLR	0.33	0.95	0.21	0.98	0.25	1.01	0.22	1.23	0.31	0.97
Raw counts + ILR	0.33	0.95	0.19	0.98	0.28	0.99	0.22	1.23	0.31	0.97
Waist circumference										
Raw counts	0.24	0.98	0.32	0.95	0.22	1.07	0.22	1.19	0.25	0.98
RA (100%)	0.24	0.98	0.33	0.95	0.23	1.08	0.27	1.09	0.28	0.97
RA + Log	0.36	0.94	0.29	0.96	0.34	0.97	0.27	1.18	0.37	0.94
Raw counts + CLR	0.39	0.93	0.28	0.96	0.34	0.97	0.27	1.19	0.37	0.94
Raw counts + ILR	0.39	0.93	0.28	0.96	0.30	0.99	0.27	1.19	0.37	0.94
Waist-hip ratio										
Raw counts	0.23	0.99	0.31	0.96	0.18	1.06	0.21	1.16	0.23	0.98
RA (100%)	0.23	0.99	0.29	0.96	0.25	1.02	0.24	1.11	0.26	0.97
RA + Log	0.34	0.94	0.26	0.97	0.29	0.99	0.28	1.16	0.35	0.95
Raw counts + CLR	0.36	0.94	0.25	0.97	0.28	1.00	0.28	1.17	0.35	0.95
Raw counts + ILR	0.36	0.94	0.25	0.97	0.28	1.00	0.28	1.17	0.35	0.95
Waist-height ratio										
Raw counts	0.21	1.00	0.31	0.95	0.22	1.06	0.20	1.23	0.23	0.98
RA (100%)	0.21	1.00	0.30	0.96	0.23	1.03	0.25	1.09	0.26	0.98
RA + Log	0.33	0.95	0.27	0.96	0.30	0.99	0.24	1.21	0.36	0.94
Raw counts + CLR	0.37	0.94	0.25	0.97	0.29	1.00	0.24	1.22	0.36	0.94
Raw counts + ILR	0.37	0.94	0.26	0.97	0.28	1.00	0.24	1.22	0.36	0.95
Body adiposity index										
Raw counts	0.13	1.02	0.14	0.99	0.13	1.07	0.10	1.28	0.13	1.00
RA (100%)	0.13	1.02	0.14	0.99	0.09	1.15	0.12	1.15	0.13	1.01
RA + Log	0.23	0.99	0.14	0.99	0.18	1.03	0.13	1.29	0.23	0.99
Raw counts + CLR	0.24	0.99	0.12	0.99	0.15	1.06	0.13	1.30	0.24	1.00
Raw counts + ILR	0.24	0.99	0.12	0.99	0.13	1.06	0.13	1.30	0.24	1.00
Fat mass index										
Raw counts	0.14	1.02	0.17	0.99	0.11	1.07	0.12	1.21	0.16	1.00
RA (100%)	0.14	1.02	0.18	0.98	0.16	1.05	0.14	1.14	0.16	1.01
RA + Log	0.26	0.98	0.15	0.99	0.18	1.04	0.17	1.26	0.26	0.98
Raw counts + CLR	0.28	0.97	0.17	0.99	0.19	1.03	0.17	1.27	0.26	0.99
Raw counts + ILR	0.28	0.97	0.16	0.99	0.17	1.05	0.17	1.27	0.26	0.99
Lean body mass index										
Raw counts	0.25	0.99	0.32	0.95	0.18	1.12	0.19	1.26	0.24	0.98
RA (100%)	0.25	0.99	0.33	0.95	0.25	1.02	0.25	1.10	0.27	0.97
RA + Log	0.34	0.94	0.27	0.96	0.30	0.99	0.28	1.18	0.33	0.96
Raw counts + CLR	0.36	0.94	0.26	0.97	0.28	1.00	0.28	1.19	0.33	0.96
Raw counts + ILR	0.36	0.94	0.28	0.96	0.29	1.00	0.28	1.19	0.33	0.96
Appendicular muscle mass index										
Raw counts	0.25	0.99	0.32	0.95	0.22	1.07	0.20	1.24	0.23	0.98
RA (100%)	0.25	0.99	0.29	0.96	0.21	1.06	0.24	1.10	0.27	0.97
RA + Log	0.34	0.94	0.26	0.97	0.29	0.99	0.28	1.17	0.33	0.96
Raw counts + CLR	0.35	0.94	0.29	0.96	0.29	1.00	0.28	1.18	0.33	0.96
Raw counts + ILR	0.35	0.94	0.28	0.96	0.32	0.98	0.28	1.18	0.33	0.96
Body fat percentage										
Raw counts	0.14	1.02	0.19	0.98	0.08	1.12	0.10	1.19	0.15	1.00
RA (100%)	0.14	1.02	0.18	0.98	0.04	1.24	0.12	1.16	0.15	1.00
RA + Log	0.23	0.97	0.16	0.99	0.18	1.04	0.18	1.25	0.26	0.99
Raw counts + CLR	0.28	0.97	0.14	0.99	0.15	1.05	0.18	1.25	0.25	1.00
Raw counts + ILR	0.28	0.97	0.12	0.99	0.19	1.04	0.18	1.25	0.26	1.00

**Table 3:** Comparison of predictive performance in study population and sex-stratified subsets: Each (sub-)set was scaled (adiposity measure) and transformed (OTUs) separately. CLR: centered log-ratio; NPK: normalized poly kernel; RA: relative abundance (100%); PLS4: partial least squares with four principal components; SVMReg: support vector machine regression.

Abdominal Adiposity Measures	Total Population (*n* = 1923)		Men (*n* = 936)		Women (*n* = 987)	
	CC	RMSE	CC	RMSE	CC	RMSE
SVMReg NPK and CLR						
Waist circumference	0.39	0.93	0.24	0.98	0.32	0.96
Waist–hip ratio	0.36	0.94	0.24	0.98	0.26	0.98
Waist–height ratio	0.37	0.94	0.26	0.97	0.34	0.95
PLS4 and RA + Log						
Waist circumference	0.37	0.94	0.27	0.99	0.31	0.98
Waist–hip ratio	0.35	0.95	0.24	1.01	0.27	1.00
Waist–height ratio	0.36	0.94	0.28	0.99	0.34	0.97

## Supplementary Materials

The following are available online at https://www.mdpi.com/2076-2607/8/4/547/s1, Figure S1: Alpha diversity as effective number of species by Simpson index in men and women; Table S1: Extended benchmark data on total study population (*n* = 1923); Table S2a. Comparison of levels of prevalence in waist–height ratio; Table S2b. Comparison of levels of relative abundance; Table S3. Comparison of pseudocounts; Table S4: OTUs.
