# Supplementary material for: Investigation of Adiposity Measures and Operational Taxonomic unit (OTU) Data Transformation Procedures in Stool Samples from a German Cohort Study Using Machine Learning Algorithms
Source: Microorganisms. 2020 Apr 10;8(4):547. doi: 10.3390/microorganisms8040547 (PMC7232268; doi:10.3390/microorganisms8040547)
Supplement: Supplementary file 1 [file microorganisms-08-00547-s001.zip › Supplementary Materials/Supplementary Materials.pdf]

Article

Investigation of Adiposity Measures and Operational Taxonomic unit (OTU) Data Transformation Procedures in Stool Samples from a German Cohort Study Using Machine Learning Algorithms

Martina Troll [1,2,*], Stefan Brandmaier [1,2], Sandra Reitmeier [3,4], Jonathan Adam [1,2], Sapna Sharma [1,2], Alice Sommer [2,5], Marie-Abèle Bind [5], Klaus Neuhaus [3], Thomas Clavel [3,6], Jerzy Adamski [7,8,9], Dirk Haller [3,4], Annette Peters [2,10] and Harald Grallert [1,2,11,*]

[1] Research Unit of Molecular Epidemiology, Helmholtz Zentrum München, 85764 Neuherberg, Germany; stefan.brandmaier@helmholtz-muenchen.de (S.B.); jonathan.adam@helmholtz-muenchen.de (J.A.); sapna.sharma@helmholtz-muenchen.de (S.S.);
[2] Institute of Epidemiology, Helmholtz Zentrum München, 85764 Neuherberg, Germany; ajsommer@fas.harvard.edu (A.S.); peters@helmholtz-muenchen.de (A.P.)
[3] ZIEL Institute for Food & Health, Technical University of Munich, 85354 Freising-Weihenstephan, Germany; sandra.reitmeier@tum.de (S.R.); neuhaus@tum.de (K.N.); tclavel@ukaachen.de (T.C.); dirk.haller@tum.de (D.H.)
[4] Chair of Nutrition and Immunology, Technical University of Munich, 85354 Freising-Weihenstephan, Germany
[5] Department of Statistics, Faculty of Arts and Sciences, Harvard University, Cambridge, MA 02138-2901 , USA; ma.bind@mail.harvard.edu
[6] Functional Microbiome Research Group, Institute of Medical Microbiology, RWTH University Hospital, 52074 Aachen, Germany
[7] Research Unit Molecular Endocrinology and Metabolism, Helmholtz Zentrum München, 85764 Neuherberg, Germany; adamski@helmholtz-muenchen.de
[8] Chair of Experimental Genetics, Technical University of Munich, 85350 Freising-Weihenstephan, Germany
[9] Department of Biochemistry, Yong Loo Lin School of Medicine, National University of Singapore, 117597 Singapore
[10] Chair of Epidemiology, Faculty of Medicine, Ludwig-Maximilians-University München, 81377 Munich, Germany
[11] German Center for Diabetes Research (DZD), 85764 Neuherberg, Germany
* Correspondence: martina.troll@helmholtz-muenchen.de (M.T.); harald.grallert@helmholtz-muenchen.de (H.G.)

Received: 9 March 2020; Accepted: 7 April 2020; Published: 10 April 2020

Supplementary Materials:

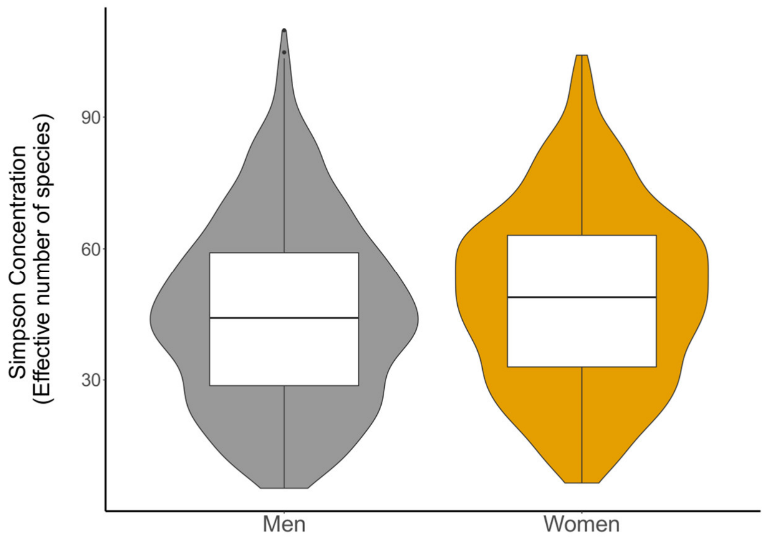

Figure S1. Alpha diversity as effective number of species by Simpson index in men and women.

Table S1: Extended benchmark data on total study population (n = 1,923).

Characteristics	Mean	SD	Median	Min	Max
Age [years]	60.0	12.1	60.0	38.0	88.0
Body mass index [kg/m^2]	27.9	5.0	27.1	18.6	62.2
Waist circumference [cm]	97.0	14.2	97.2	64.7	172.3
Waist-hip ratio	0.91	0.09	0.91	0.65	1.20
Waist-height ratio	0.58	0.08	0.57	0.39	0.95
Body adiposity index	31.0	6.0	29.7	19.2	61.4
Fat mass index [kg/m^2]	9.4	3.4	8.8	2.6	29.2
Lean body mass index [kg/m^2]	18.5	2.6	18.4	12.8	33.0
Appendicular muscle mass index [kg/m^2]	7.7	1.3	7.6	4.8	14.6
Body fat [%]	32.9	7.1	32.8	11.9	50.9

Table S2a. Comparison of levels of prevalence in waist-height ratio. In each step, OTUs present in less than x % of the samples were excluded. For each subset separately, CLR relative abundance (RA) and log-transformation with one pseudocount was used as prior data transformation. Support vector machine regression (SVMReg) with normalized poly kernel (NPK) and Partial Least Squares (PLS) with 10-fold cross-validation was used. CC: correlation coefficient; RMSE: root mean squared error.

**Excluded percentage missingness in samples**	**Number of OTUs**	**SVMReg NPK & CLR**		**PLS4 & RA+ Log**	
		CC	RMSE	CC	RMSE
0 %	2,089 (100 %)	0.37	0.94	0.36	0.94
10 %	958 (46 %)	0.37	0.94	0.36	0.94
20 %	766 (37 %)	0.36	0.94	0.36	0.94
30 %	591 (28 %)	0.34	0.95	0.36	0.94
40 %	451 (22 %)	0.34	0.95	0.35	0.94
50 %	359 (17 %)	0.32	0.96	0.34	0.95
60 %	276 (13 %)	0.30	0.97	0.33	0.95
70 %	200 (10 %)	0.26	0.99	0.30	0.96
80 %	122 (6 %)	0.21	1.01	0.28	0.96
90 %	52 (2 %)	0.19	1.01	0.24	0.97

Table S2b. Comparison of levels of relative abundance. In each step, OTUs with an across-sample relative abundance of x % were excluded. For each subset separately, CLR or relative abundance (RA) and log-transformation with one pseudocount was used as prior data transformation. Support vector machine regression (SVMReg) with normalized poly kernel (NPK) and Partial Least Squares (PLS) with 10-fold cross-validation was used. CC: correlation coefficient; RMSE: root mean squared error.

**Min. relative abundance in samples**	**Number of OTUs**	**SVMReg NPK & CLR**		**PLS4 & RA+ Log**	
		CC	RMSE	CC	RMSE
0.0 %	2,089 (100 %)	0.37	0.94	0.36	0.94
0.001 %	1,917 (92 %)	0.37	0.94	0.36	0.94
0.005 %	1,353 (65 %)	0.37	0.94	0.36	0.94
0.01 %	1,064 (51 %)	0.36	0.94	0.36	0.94
0.05 %	384 (18 %)	0.30	0.97	0.34	0.95
0.1 %	204 (10 %)	0.27	0.98	0.31	0.96
0.5 %	28 (1 %)	0.14	1.02	0.22	0.98

Minimal effects of zero replacement via pseudocounts

The handling of zeros is crucial in an analytical approach that uses log transformations. One approach is to add a certain value to all measurements in the data set and therefore offset all zero values, so called pseudocounts. Since there is no common answer to the question of optimal pseudocount value choice [1] we evaluated the performance of support vector machine in waist-height ratio prediction according to varying pseudocount values. Previous studies have used very small values to offset zeros in the data set,

e.g. 10^{-6} [2,3]. In our study, the value of the pseudocount was only minimally relevant to the prediction performance within the same machine learning algorithm (Table S3). However, using the value one leads to the retaining of zeros after the log transformation, therefore preserving the original data structure.

Table S3. Comparison of pseudocounts. CLR with varying pseudocounts was used as prior data transformation. Support vector machine regression (SVMReg) with normalized poly kernel (NPK) and 10-fold cross-validation was used. Waist-height ratio was used as dependent variable. CC: correlation coefficient; RMSE: root mean squared error.

**Value of pseudocount**	**SVMReg NPK**	
	CC	RMSE
1	0.37	0.94
0.1	0.38	0.93
0.01	0.38	0.93
0.000001	0.38	0.93

References

1. Weiss, S.; Xu, Z.Z.; Peddada, S.; Amir, A.; Bittinger, K.; Gonzalez, A.; Lozupone, C.; Zaneveld, J.R.; Vázquez-Baeza, Y.; Birmingham, A.; et al. Normalization and microbial differential abundance strategies depend upon data characteristics. *Microbiome* **2017**, *5*, 27.
2. Beaumont, M.; Goodrich, J.K.; Jackson, M.A.; Yet, I.; Davenport, E.R.; Vieira-Silva, S.; Debelius, J.; Pallister, T.; Mangino, M.; Raes, J.; et al. Heritable components of the human fecal microbiome are associated with visceral fat. *Genome Biol.* **2016**, *17*, 189.
3. Zierer, J.; Jackson, M.A.; Kastenmüller, G.; Mangino, M.; Long, T.; Telenti, A.; Mohney, R.P.; Small, K.S.; Bell, J.T.; Steves, C.J.; et al. The fecal metabolome as a functional readout of the gut microbiome. *Nat. Genet.* **2018**, *50*, 790–795.
