# Supplementary figures and images for: Investigation of Adiposity Measures and Operational Taxonomic unit (OTU) Data Transformation Procedures in Stool Samples from a German Cohort Study Using Machine Learning Algorithms
Source: Microorganisms. 2020 Apr 10;8(4):547. doi: 10.3390/microorganisms8040547 (PMC7232268; doi:10.3390/microorganisms8040547)

Simpson Concentration  
(Effective number of species)

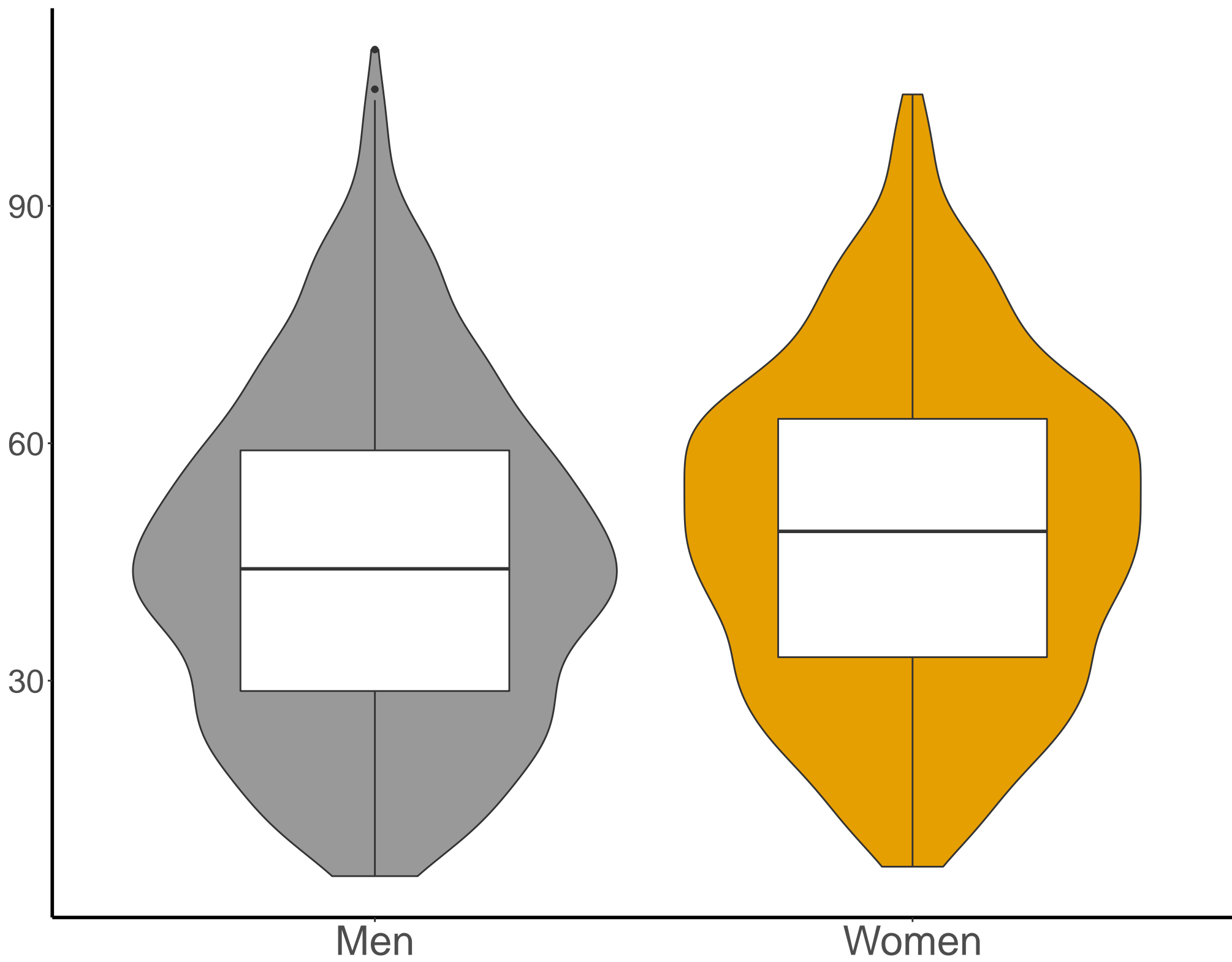

Supplement: Supplementary file 1 [file microorganisms-08-00547-s001.zip › Supplementary Materials/FigureS1.pdf]
